# Effects of black adzuki bean (*Vigna angularis*, Geomguseul) extract on body composition and hypothalamic neuropeptide expression in rats fed a high-fat diet

**DOI:** 10.3402/fnr.v59.27719

**Published:** 2015-10-21

**Authors:** Mina Kim, Seok-Bo Song, Youn-Soo Cha

**Affiliations:** 1 Department of Food Science and Human Nutrition, BK21 PLUS Program, Chonbuk National University, Jeonju, Republic of Korea; 2 Department of Functional Crops, National Institute of Crop Science, Rural Development Administration, Miryang, Republic of Korea

**Keywords:** *Vigna angularis*, obesity, hyperlipidemia, hypothalamic neuropeptides, POMC, CART, ObRb, AGRP

## Abstract

**Background:**

Obesity is often considered to result from either excessive food intake or insufficient physical activity. Adzuki beans have been evaluated as potential remedies for various health conditions, and recent studies have reported their effects on the regulation of lipid metabolism, but it remains to be determined whether they may be effective in overcoming obesity by regulating appetite and satiety.

**Objective:**

This study investigated the effect of black adzuki bean (BAB) extract on body composition and hypothalamic neuropeptide expression in Sprague Dawley rats (Rattus norvegicus) fed a high-fat diet.

**Design:**

The rats were fed for 8 weeks with a control diet containing 10 kcal% from fat (CD), a high-fat diet containing 60 kcal% from fat (HD), or a high-fat diet with 1% or 2% freeze-dried ethanolic extract powder of BAB (BAB-1 and BAB-2).

**Results:**

The body weights and epididymal fat weights were significantly reduced and the serum lipid profiles were improved in the group fed the diet containing BAB compared to the HD group. The expression of *AGRP* mRNA significantly decreased in the BAB groups, and treatment with BAB-2 resulted in a marked induction of the mRNA expression of *POMC* and *CART*, which are anorexigenic neuropeptides that suppress food intake. Furthermore, mRNA expression levels of *ObRb*, a gene related to leptin sensitivity in the hypothalamus, were significantly higher in the BAB groups than in the HD group.

**Conclusions:**

These results suggest that supplementation with BAB has a significant effect on body weight via regulation of hypothalamic neuropeptides.

Obesity is often considered to result from either excessive food intake or insufficient physical activity. We believe that obesity can best be viewed in terms of energy balance (1). Energy balance is mediated through endocrine and neural signals coming from a variety of peripheral tissues to the central nervous system (CNS) (2). Genes and proteins that play roles in obesity may regulate appetite/satiety and energy storage/expenditure in adipose tissue (2, 3).

Natural anti-obesity treatments can induce weight loss through several mechanisms. Some medicinal plants prevent lipid absorption and adipogenesis, increase the metabolic rate (which, in turn, enhances thermogenesis and lipolysis), or suppress appetite and induce satiety (4). Adzuki beans have been evaluated as potential remedies for hypercholesterolemia, hyperglycemia, and inflammation in mice and rats (5–8), and preliminary data have shown that black adzuki beans (BAB) inhibit proliferation and mitotic clonal expansion and subsequently inhibit the adipogenesis of 3T3-L1 cells (9). Although recent studies have reported evidence that the adzuki beans affect the regulation of lipid metabolism, it remains to be determined whether they may be effective in overcoming obesity by regulating appetite and satiety. Therefore, we considered the connection between body weight and neuropeptide expression in the hypothalamus. This study provides novel evidence that the reduced weight gain of rats consuming BAB extract could be accomplished through the regulation of the expression of hypothalamic orexigenic and anorexigenic neuropeptides.

## Materials and methods

### Preparation and composition of test material

BAB extract was obtained according to a previously reported method (9). In brief, BAB (*Vigna angularis*, Geomguseul) harvested by the National Institute of Crop Science, Rural Development Administration in the Republic of Korea, was ground, extracted by adding 80% ethanol, concentrated through evaporation of the ethanol with a rotary vacuum evaporator (Eyela, Tokyo, Japan), and freeze-dried. It was stored at −20°C before use. The composition per 100 g of freeze-dried powdered sample of the BAB was as follows: 76.7 g carbohydrate, 0.7 g protein, 8 g lipid, 3.3 g ash, 0.7 g fiber, and 0.5 g total flavonoids.

### Animal study design and experiments

Four-week-old male Sprague Dawley rats (Rattus norvegicus) (*n*=24) obtained from Japan SLC, Ltd. (Hamamatsu, Japan), were used in the study. The rats were housed in plastic cages with controlled conditions (12-h light/dark cycle at 22±2°C). After 1 week, they were randomly divided into four groups of six animals each, and were fed for 8 weeks with either a control diet containing 10 kcal% from fat (CD), a high-fat diet containing 60 kcal% from fat (HD), or a high-fat diet with 1% or 2% freeze-dried powder of BAB ethanolic extract (BAB-1 and BAB-2). The compositions of the diets are listed in Table 1; these were based on D12450B and D12492 (Research Diets, Inc., New Brunswick, NJ, USA). Rats from all groups were then given unlimited access to the diet and water. Body weight was recorded once a week. On the final day of the experiment, after a 12-h fast, the rats were sacrificed by ethyl ether anesthesia, and the blood was immediately collected separately using a polyethylene tube with or without heparin, DPP-IV, and aprotinin to measure ghrelin, leptin, glucagon, triglyceride (TG), total cholesterol (TC), and high-density lipoprotein cholesterol (HDL-C) levels. The brain, liver, and epididymal fat were excised and frozen immediately in liquid nitrogen. These samples were kept at −80°C until analysis. The experimental animals were handled in accordance with the Guide for the Care and Use of Laboratory Animals after obtaining the approval of the Institutional Animal Care and Use Committees of the Chonbuk National University (CBU 2014-00035).

**Table 1:** Composition of experimental diets

	Control diet	High-fat diet		
		
Component (g/100g)	CD	HD	BAB-1	BAB-2
Casein	18.96	25.84	25.59	25.33
Cystine	0.28	0.39	0.38	0.38
Corn starch	29.86	–	–	–
Maltodextrin	3.32	16.15	15.99	15.83
Sucrose	33.17	8.89	8.80	8.71
Cellulose	4.74	6.46	6.40	6.33
Soybean oil	2.37	3.23	3.20	3.17
Lard	1.90	31.66	31.34	31.03
Mineral	0.95	1.29	1.28	1.27
Dicalcium phosphate	1.23	1.68	1.66	1.65
Calcium carbonate	0.52	0.71	0.70	0.70
Potassium citrate	1.56	2.13	2.11	2.09
Vitamin mix	0.95	1.29	1.28	1.27
Choline bitartrate	0.19	0.26	0.26	0.25
Black adzuki beans			1	2
kcal/g	3.95	5.24	5.24	5.23

Dried powdered black adzuki beans were extracted three times with 80% ethanol and evaporated under vacuum using a rotary evaporator. CD, control diets containing 10 kcal% fat (D12450B); HD, high-fat diets control containing 60 kcal% fat (D12492); BAB-1 and BAB-2, high-fat diets (D12492) plus 1% or 2% freeze-dried ethanolic extract of black adzuki beans.

### Food intake

Food intake was recorded three times a week. Each group consisted of six rats, housed in two cages with three animals in each. The mean of the total food intake during one week was used for statistical analysis. The percent reduction in food intake (g/day) (%FIR) was calculated using the following equation (10): %FIR=[food intake (HD) – food intake (BAB)]×100/food intake (HD).

### Quantification of serum metabolic parameters, leptin, and ghrelin

The profiles of serum TG, TC, and HDL-C were measured using a clinical chemistry analyzer (Dri-Chem 3500, Fuji Film Co., Tokyo, Japan), while ghrelin (RayBiotech, Inc., Norcross, GA, USA) and leptin (R&D Systems, Minneapolis, MN, USA) levels were analyzed using commercial assay kits, as per the manufacturers’ instructions. The non-HDL-C concentration was calculated as follows: [non-HDL-C]=[TC] – [HDL-C].

### Quantitative real-time polymerase chain reaction analysis

Hypothalami were dissected from the brains, and RNA was extracted and reverse-transcribed into cDNA using a high-capacity cDNA reverse transcription kit (Applied Biosystems, Foster City, CA, USA). Then, the RNA expression level was quantified by quantitative real-time PCR using SYBR Green PCR Master Mix (Applied Biosystems, Woolston, Warrington, UK) and the 7500 Real Time PCR system (Applied Biosystems, Foster City, CA, USA). The gene-specific primers used are given in Table 2. Relative quantification was achieved using the ΔΔCt method.

**Table 2:** Oligonucleotide primers used for quantitative real-time PCR

Gene	GenBank accession number	Primer sequences (5’–3’)	Orientation
*NPY*	NM012614	GCTAGGTAACAAACGAATGGGG	Forward
		CACATGGAAGGGTCTTCAAGC	Reverse
*AGRP*	XM574228	GCAAGGATCAACAAGCAA	Forward
		GAACAGGGCCTGGTCAGA	Reverse
*POMC*	NM139326	TCCGAGAAGAGCCAGACG	Forward
		GCCTTGGAGTGAGAAGACCC	Reverse
*CART*	NM017110	GAGCCCTGGACATCTACTC	Forward
		ATCGGAATGCGTTTACTC	Reverse
*MC4R*	NM013099	ATGAACTCCACCCACCAC	Forward
		CATAGCATCCTCCGTCTG	Reverse
*ObRb*	U52966.1	GGGAACCTGTGAGGATGAGTGT	Forward
		TTTCCACTGTTTTCACGTTGCT	Reverse
*GAPDH*	NM17701	CAGTGCCAGCCTCGTCTCATA	Forward
		TGCCGTGGGTAGAGTCATA	Reverse

### Statistical analyses

All data are presented as mean±SD. Statistical significance was determined using one-way ANOVA, and differences between the means were assessed using Duncan's multiple range test; *p*<0.05 was considered significant.

## Results

### Body composition and food intake

**Fig. 1:**
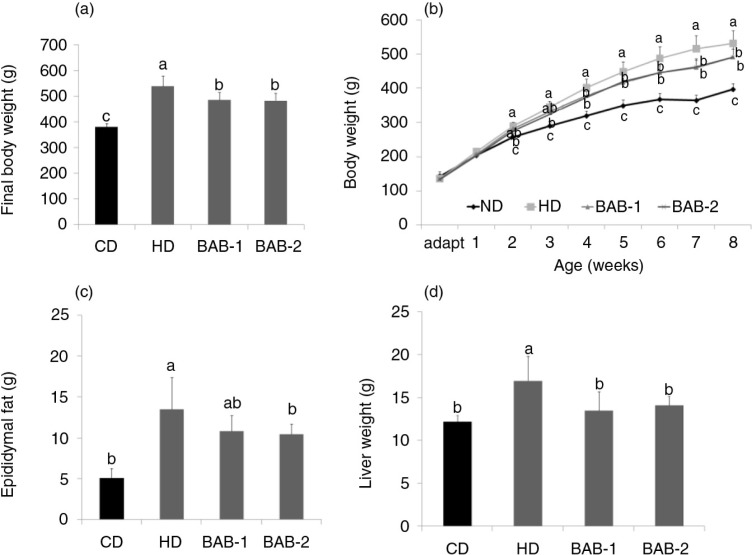
Effect of black adzuki beans on (a, b) body weight and (c, d) organ weight in rats. Rats were divided into four groups of six rats each. CD, control diets containing 10 kcal% fat (D12450B); HD, high-fat diets control containing 60 kcal% fat (D12492); BAB-1 and BAB-2, high-fat diets (D12492) plus 1% or 2% (W/W) freeze-dried ethanolic extract of black adzuki beans. Data are expressed as mean±SD with different letters indicating a significant difference among groups, according to ANOVA with Duncan's multiple range test (*p*<0.05).

As shown in Fig. 1, there were significant differences in body weight gain in rats fed the different diets throughout the study. Rats in the HD group gained more weight than those in the CD and BAB groups, and the final body weights were significantly higher in the HD group (539.38±39.92 g) than in the CD group (381.15±13.83 g). BAB treatment caused a significant decrease in body weight (BAB-1: 486.38±29.11 g, BAB-2: 483.25±28.33 g).

In agreement with the reduced body weights of BAB-fed rats, significant reductions in epididymal fat and liver weight were observed in the BAB-containing diet groups compared to the HD group.

**Fig. 2:**
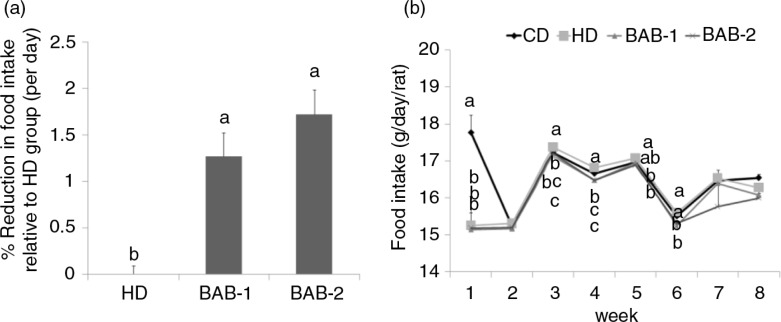
Effect of black adzuki beans on percent (a) reduction in food intake and (b) weekly food intake in rats. The reduction in food intake (%FIR) was calculated relative to HD group. Rats were divided into four groups of six rats each. CD, control diets containing 10 kcal% fat (D12450B); HD, high-fat diets control containing 60 kcal% fat (D12492); BAB-1 and BAB-2, high-fat diets (D12492) plus 1% or 2% (W/W) freeze-dried ethanolic extract of black adzuki beans. Data are expressed as mean±SD with different letters indicating a significant difference among groups, according to ANOVA with Duncan's multiple range test (*p*<0.05).

Food intake in BAB-fed rats was significantly lower than that recorded for HD rats (Fig. 2b). The suppression of food intake by BAB persisted intermittently; as a result, there were 1.27% (BAB-1) or 1.72% (BAB-2) reductions in food intake relative to that of HD control rats (Fig. 2a).

### Serum lipid parameters

A marked increase in the serum lipid concentrations was found in rats fed the HD compared to those fed the CD (Table 3). The TG levels were significantly lower in the BAB groups than in the HD group. The TC level was significantly lower in the BAB-1 group than in the HD group and the BAB-2 group displayed a tendency of reduced TC. The HDL-C concentration increased in the BAB-2 and HD groups, but the non-HDL-C concentration substantially increased in the HD group, whereas it markedly decreased (by about 43%) in the BAB groups.

**Table 3:** Serum lipid profile in rats fed a high-fat diet containing black adzuki beans

	Control diet	High-fat diet		
		
Parameter (mg/dL)	CD	HD	BAB-1	BAB-2
Triglyceride	74.25±11.95^b^	173.63±42.11^a^	112.50±20.63^b^	113.63±33.03^b^
Total cholesterol	59.40±8.96^bc^	84.40±5.08^a^	55.00±9.08^c^	72.40±16.13^ab^
HDL cholesterol	40.40±6.02^b^	51.10±6.73^a^	35.80±9.36^b^	53.40±6.23^a^
Non-HDL cholesterol	19.00±6.02^b^	33.30±6.73^a^	19.20±9.36^b^	19.00±6.23^b^

Rats were divided into four groups of six rat each. CD; control diets containing 10 kcal% fat (D12450B), HD; high-fat diets control containing 60 kcal% fat (D12492), BAB-1 and BAB-2; high-fat diets (D12492) plus 1% or 2% (W/W) freeze-dried ethanolic extract of black adzuki beans. Data are expressed as mean±SD with different letters in the row indicating a significant difference among groups, according to ANOVA with Duncan's multiple range test (*p*<0.05).

### Plasma leptin and ghrelin levels

**Fig. 3:**
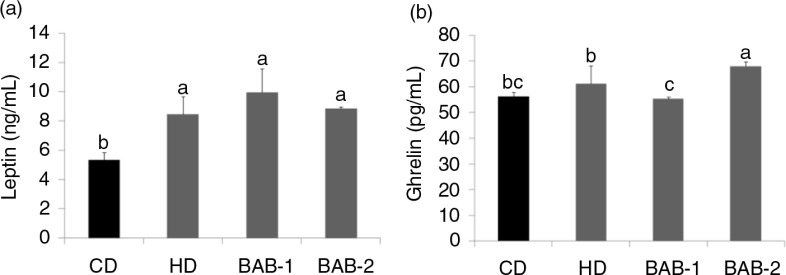
Effects of black adzuki beans on plasma (a) leptin and (b) ghrelin in rats fed a high-fat diet. Rats were divided into four groups of six rats each. CD, control diet containing 10 kcal% fat (D12450B); HD, high-fat diet control containing 60 kcal% fat (D12492); BAB-1 and BAB-2, high-fat diets (D12492) plus 1% or 2% (W/W) freeze-dried ethanolic extract of black adzuki beans. Data are expressed as mean±SD with different letters indicating a significant difference among groups, according to ANOVA with Duncan's multiple range test (*p*<0.05).

Leptin is an adipose tissue–derived hormone that suppresses appetite by inhibiting neuropeptide Y (*NPY*); on the other hand, ghrelin has been recognized as a food-intake stimulator that induces appetite by increasing the expression of *NPY* and subsequently agouti-related protein (*AGRP*) (11–14). Fasting leptin levels showed a rising tendency in the BAB-1 group, but no significant differences were found among the high-fat-fed groups (Fig. 3). Leptin levels were significantly higher in the high-fat-fed groups than in the CD group. Acetylated ghrelin levels were lower in the BAB-1 group than in the other groups.

### Appetite-related neuropeptide mRNA expression in the hypothalamus

To ascertain whether BAB had altered the levels of neuropeptides known to regulate food intake, the mRNA levels of *NPY*, *AGRP*, pro-opiomelanocortin (*POMC*), cocaine- and amphetamine-responsive transcript (*CART*), melanocortin-4 receptor (*MC4R*), and leptin receptor (*ObRb*) were investigated in the hypothalami of BAB-fed rats. The hypothalamus is an important brain region regulating energy balance via neuropeptides produced by the arcuate nucleus (ARC): *NPY* and *AGRP* (orexigenic neuropeptides) and *POMC* and *CART* (anorexigenic neuropeptides) (15).

**Fig. 4:**
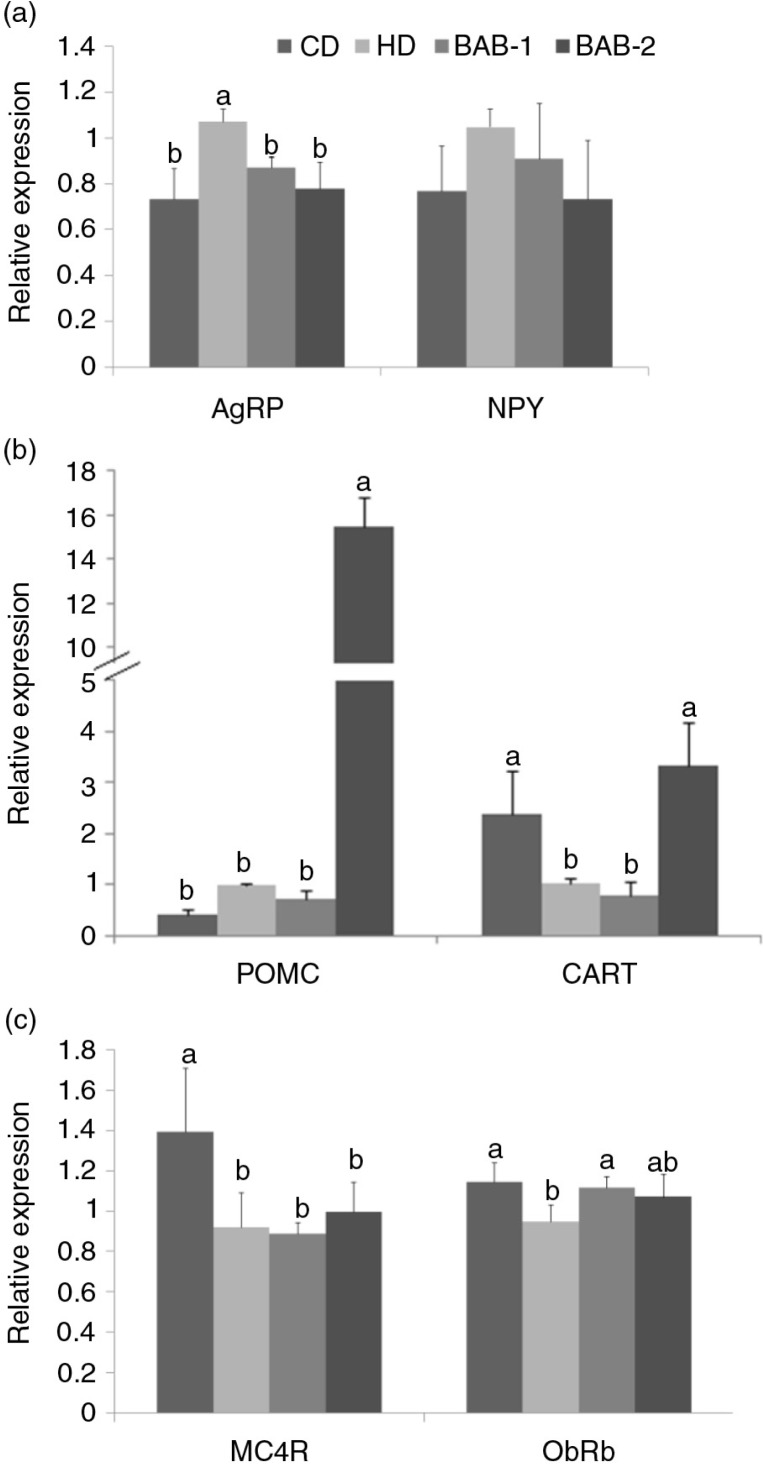
Effects of black adzuki beans on mRNA expression of hypothalamic neuropeptides in rats fed a high-fat diet. (a) Orexigenic neuropeptide, (b) anorexigenic neuropeptide, and (c) neuropeptide receptor mRNA expression were measured by quantitative real-time PCR. The mRNA expression was normalized using *GAPDH* and shown by relative expression of neuropeptide in the HD group. Data are expressed as mean±SD with different letters indicating a significant difference among groups, according to ANOVA with Duncan's multiple range test (*p*<0.05).

The expression of hypothalamic *AGRP* mRNA was reducedsignificantly in the BAB groups, and *NPY* expression showed a non-significant trend towards reduction in the BAB groups compared to the HD group (Fig. 4a). In contrast, treatment with BAB-2 resulted in a marked induction of the mRNA expression of *POMC* and *CART*, which are anorexigenic neuropeptides that suppress food intake (Fig. 4b). The mRNA expression levels of neuropeptide-related receptors also have been implicated in the process (Fig. 4c). In order to assess leptin sensitivity in the hypothalamus, we measured the mRNA expression levels of the long-form leptin receptor, *ObRb*. mRNA expression levels of *ObRb* were significantly higher in the BAB groups than in the HD group. The *MC4R* gene can control energy homeostasis by integrating signals in the hypothalamus (16), but BAB did not affect the levels of *MC4R* expression in the high-fat diet groups.

## Discussion

The anti-obesity effects of many natural products involve diverse mechanisms, including the regulation of appetite, adipogenesis, and lipolysis (17, 18). Several studies have reported that treatments with adzuki beans improved the symptoms of obesity and obesity-related diseases (5, 19, 20). Recently, we demonstrated that BAB regulated the early stages of adipocyte differentiation and, as a result, suppressed lipid accumulation and regulated many genes for adipogenesis in 3T3-L1 cells (9). Nevertheless, the mechanism by which adzuki beans exert anti-obesity effects remains unclear, and it needs to be resolved whether they may be effective in overcoming obesity by regulating appetite and satiety. In this study, an extract of BAB was added to the diet of rats, and we demonstrated that BAB prevented diet-induced obesity by regulating hypothalamic neuropeptides.

In the present study, BAB prevented diet-induced obesity and led to remarkable reductions in body weight. The results showed that the elevated weight of the epididymal fat in the HD group was significantly reduced with BAB. At the same time, along with body weight loss, BAB markedly reduced TG and non-HDL-C levels in rats fed a high-fat diet. Similar data were generated in a previous study, in which rats fed a high-fat diet containing adzuki beans displayed improved lipid profiles (19), but interestingly our study indicated that BAB also affected food intake. Thus, we reasoned that BAB might influence appetite through effects in the hypothalamus. A series of experiments were conducted to test this hypothesis.

Neurons in the hypothalamus have critical roles in the control of food intake and body weight through the orexigenic and anorexigenic actions of the neuropeptides they express (15). Two main neuronal populations exist in the hypothalamic ARC: the orexigenic *NPY/AGRP* neurons and the anorexigenic *POMC/CART* neurons (15). With this in mind, to elucidate the underlying mechanism of the appetite-reducing effects of BAB, we measured the plasma levels of leptin and ghrelin, as well as neuropeptide expression in the hypothalamus.

Leptin, a product of the OB (leptin) gene, integrates the status of peripheral fat stores with the central control of food intake for the homeostatic control of body weight by regulating the hypothalamic expression of appetite-related genes (21–23). In the ARC, *POMC* expression is increased by leptin, whereas *AGRP* and *NPY* levels are reduced (24). Leptin, an anorexic hormone, positively regulates *POMC/CART* to activate satiety and inhibit the *NPY/AGRP* pathway through specific receptors located in the hypothalamus (22, 25, 26). These changes in neuropeptide expression serve to decrease food intake and enhance weight loss (26). On the other hand, the fasting state serves to augment ghrelin (27), which induces appetite by increasing the expression of *NPY*, a potent stimulator of the hunger sensation, and subsequently *AGRP* (11–13). Based on these mechanisms of action, ghrelin and leptin exert antagonistic effects via their specific receptors in the CNS (12, 13).

In this study, the food intake ratio (%FIR) was significantly reduced in the BAB treatment groups (Fig. 2). Additionally, the mRNA expression of orexigenic neuropeptides such as *AGRP* was significantly reduced in the BAB treatment groups compared to the HD group, while mRNA expression levels of the anorexigenic *POMC* and *CART* neuropeptides were significantly increased in the BAB groups (Fig. 4). Leptin levels were similar among the high-fat diet fed groups, despite of BAB treatment, whereas we found that the mRNA expression levels of *ObRb* were significantly higher in the BAB groups than in the HD group. Thus, BAB can stimulate the action of leptin through increased leptin sensitivity, as well as block the action of ghrelin. The action of leptin in regulating neuroendocrine function is mediated in the brain by its receptor, *ObRb* (28). Defective leptin signaling, due to either leptin deficiency or mutations in the leptin receptor, leads to the development of obesity (22). Therefore, our studies indicate that BAB treatment alters hypothalamic neuropeptides in a way that improves symptoms of obesity.

In summary, several lines of evidence support the conclusion that BAB acts as a regulator of the action of hypothalamic leptin. First, BAB improved leptin sensitivity by increasing the mRNA expression levels of *ObRb*. Second, leptin increased *POMC/CART* mRNA levels to activate satiety and inhibit *NPY/AGRP*. Third, BAB treatment significantly reduced the food intake ratio (%FIR). Accordingly, BAB improved the lipid profiles and affected the epididymal fat weights and body weights of high-fat fed rats.

## Conclusions

This study shows the effect of BAB on body composition with regard to hypothalamic neuropeptide expression in rats fed a high-fat diet. BAB helps in controlling diet-induced obesity through improving lipid profile and decreasing epididymal fat weight and body weight. Furthermore, BAB might improve leptin sensitivity through increased the mRNA expression levels of *ObRb*. Consequently, leptin can increase *POMC/CART* and inhibit the *NPY/AGRP*. Interestingly, the food intake ratio (%FIR) was also significantly decreased in BAB treatment groups. These results suggest that supplementation with BAB has a significant effect on body weight via regulation of hypothalamus neuropeptides.
